# A novel *MYORG* mutation causes primary familial brain calcification with migraine: Case report and literature review

**DOI:** 10.3389/fneur.2023.1110227

**Published:** 2023-02-02

**Authors:** Tingwei Song, Yuwen Zhao, Guo Wen, Juan Du, Qian Xu

**Affiliations:** ^1^Department of Neurology, Xiangya Hospital, Central South University, Changsha, China; ^2^National Clinical Research Center for Geriatric Disorders, Xiangya Hospital, Changsha, China; ^3^Key Laboratory of Hunan Province in Neurodegenerative Disorders, Central South University, Changsha, China

**Keywords:** primary familial brain calcification, novel *MYORG* mutation, migraine, case report, literature review

## Abstract

Primary familial brain calcification (PFBC) is a disorder in which pathologic calcification of the basal ganglia, cerebellum, or other brain regions with bilateral symmetry occurs. Common clinical symptoms include dysarthria, cerebellar symptoms, motor deficits, and cognitive impairment. Genetic factors are an important cause of the disease; however autosomal recessive (AR) inheritance is rare. In 2018, the myogenesis-regulated glycosidase (*MYORG*) gene was the first to be associated with AR-PFBC. The present case is a 24-year-old woman with AR-PFBC that presented with migraine at the age of 16 years. Symmetrical patchy calcifications were seen in the bilateral cerebellopontine nuclei, thalamus, basal ganglia, and radiocoronal area on computed tomography and magnetic resonance imaging. AR-PFBC with migraine as the main clinical symptom is rare. Whole-exome sequencing revealed a compound heterozygous mutation in the *MYORG* gene, one of which has not been previously reported. Our case highlights the pathogenic profile of the *MYORG* gene, and demonstrates the need for exclusion of calcium deposits in the brain for migraine patients with AR inheritance.

## Introduction

Primary familial brain calcification (PFBC, OMIM #213600) is a disorder in which the main pathological finding is calcification of the basal ganglia, cerebellum, or other brain regions with bilateral symmetry (1–3). Genetic components is an important cause of PFBC, which can exhibit both autosomal dominant (AD) and autosomal recessive (AR) inheritance. Autosomal recessive-primary familial brain calcification (AR-PFBC) generally has a higher clinical penetrance and a more severe pattern of calcifications compared to autosomal dominant-primary familial brain calcification (AD-PFBC) (1, 3, 4). The genes involved in AD-PFBC include *SLC20A2* (NM_001257180.2), *PDGFB* (NM_002608.4), *PDGFRB* (NM_001355016.2), and *XPR1* (NM_001135669.2), which are associated with calcium and phosphorus metabolism and the blood-brain barrier (5–7). Instead, genes involved in AR-PFBC include *MYORG* (NM_020702.5) and *JAM2* (NM_001270407.2). The myogenesis-regulated glycosidase (*MYORG*) gene was first identified in 2018 (8). The typical indications of AR-PFBC are chronic progressive motor impairment, cognitive impairment, dysarthria, and cerebellar symptoms, whereas migraine presentation is rare (8–10). Here we report the case of a 24-year-old woman with AR-PFBC and migraine onset at the age of 16 years harboring *MYORG* compound heterozygous variants, one of which has not been previously reported.

## Case presentation

A 24-year-old Chinese woman was admitted to our outpatient clinic with migraine headache, reportedly experienced for 8 years. She first developed recurrent episodes of headache at the age of 16, associated with no apparent trigger. These symptoms occurred one to two times a year, lasted for 1–2 days at a time, and resolved spontaneously. The profession of the patient was a middle school art teacher, and her academic performance during her school period had remained good, with no history of psychological illness or psychiatric disorders. The patient was born to non-consanguineous parents, and her physical examination showed no abnormality except for minor knee and bicep brisk reflexes.

**Figure 1:**
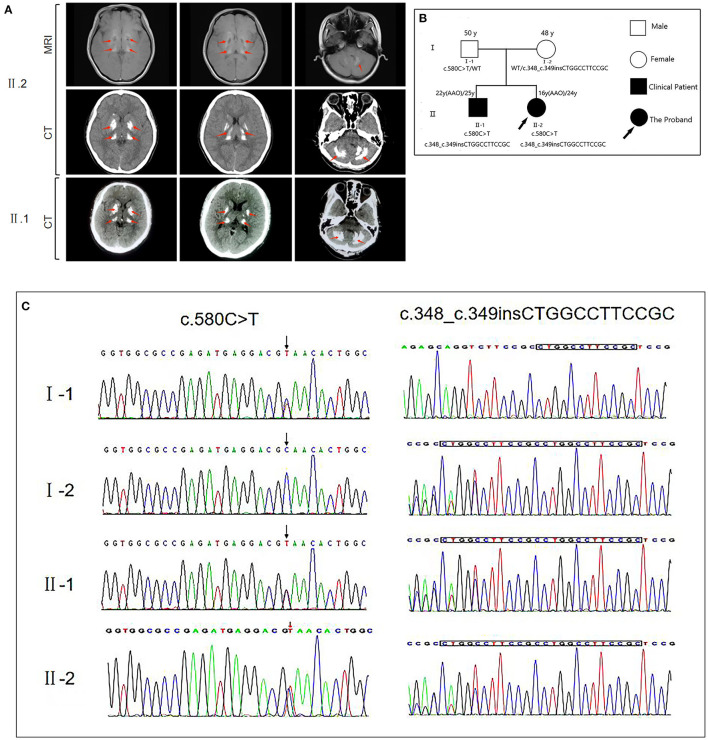
**(A)** Brain magnetic resonance imaging (MRI) and computed tomography (CT) for the proband (II.2) reveal bilateral calcification in the basal ganglia, thalamus, cerebellum, and pons. CT of the proband's brother (II.1) reveals bilateral calcification in the basal ganglia, thalamus, and cerebellum. The calcification sites showed a high degree of similarity between the patients. **(B)** The genetic pedigree for this case. **(C)** *MYORG* gene sequencing of the family members of this case. FM, family; AAO, age at onset; WT, wild type.

The patient's endocrine examination showed normal serum allosteric parathyroid hormone, calcium, and phosphorus levels. Moreover, the results of the Foaming experimental and right heart contrast echocardiography were normal. Computed tomography (CT) and magnetic resonance imaging revealed symmetrical patchy calcifications in the bilateral cerebellopontine nuclei, thalamus, basal ganglia, and radiocoronal areas (Figure 1). The patient had a Montreal Cognitive Assessment score of 26 (delayed memory minus 4 points did not meet the diagnostic criteria for cognitive dysfunction), and a Mini-Mental State Examination score of 28. The patient also had a migraine-specific Quality of Life Questionnaire score of 21, and a Headache Impact Test-6 score of 51. We administered Oxiracetam and nicergoline for neurotrophic treatment and improvement of cerebrovascular circulation, and the patient reported symptom improvement. The patient's brother had also undergone CT examination at another hospital due to occasional headache, which revealed multiple symmetrical calcifications in the bilateral basal ganglia, dorsal thalamus, cerebellar hemispheres, and vermis. The calcification sites and morphology of both siblings exhibited a high degree of similarity (Figure 1).

We then performed whole-exome sequencing on the patient, identifying that the proband had potential compound heterozygous variants in the *MYORG* gene, including two heterozygous variants, namely c.348_c.349insCTGGCCTTCCGC (p.116_117insLAFR) and c.580G>T (p.Q194^*^) that were confirmed by Sanger sequencing (Figure 1). The insertion c.348_c.349insCTGGCCTTCCGC variant is a known pathogenic mutation (8). However, the loss-of-function c.580G > T *MYORG* gene variant has not been reported in ClinVar, and no publication has reported this mutation so far (1–3, 8, 11). Both variants were predicted to be damaging by multiple *in silico* prediction tools, including SIFT, Polyphen2, CADD, and MutationTaster. Additionally, the novel mutant (c.580G > T) is not listed in the 1,000 Genomes, NHLBI GO Exome Sequencing Project, and Exome Aggregation Consortium databases. According to the American College of Medical Genetics guidelines, the non-sense c.580G > T mutant is considered “potentially pathogenic: PVS1-Strong + PM2,” whereas the insertional c.348_c.349insCTGGCCTTCCGC mutant is considered “possibly pathogenic: PM3-Strong + PM4.” With consent, we also performed whole-exome sequencing on the patient's parents and brother. We found that the father carried only the non-sense c.580G > T variant, while the mother carried only the insertion c.348_c.349insCTGGCCTTCCGC variant. The brother carried both variants identified in the proband. Collectively, this information confirmed the two variants as the compound heterozygous variants in the *MYORG* gene, and as pathogenic *MYORG* variants associated with PFBC.

## Discussion

Autosomal recessive-primary familial cerebral calcification (AR-PFBC) is caused by mutations in the *MYORG* gene, and has an age of onset between 38 and 53 years (1). The typical indications of AR-PFBC and of the *MYORG* variant are: verbal deficits, chronic progressive motor deficits, ataxia, cognitive deficits, and psychiatric symptoms (8, 10, 12). Among them, verbal impairment is often the first symptom of PFBC due to a *MYORG* variant (12, 13), while cognitive impairment is usually milder in AR-PFBC than in AD-PFBC and does not lead to major neurocognitive impairment (8, 14). Migraine as the main symptom is rare in AR-PFBC, with only one similar case having been reported in a 12-year-old Turkish girl with AR-PFBC (10). Cognitive deficits and depression have been documented as the main non-motor symptoms and signs of PFBC; instead headache is less common, and is observed in ~8% of cases (1).

Here, we report the case of a 24-year-old female whose symptoms began at the age of 16 years. The patient reported experiencing migraines over the last 8 years, together with some other cognitive symptoms but without motor symptoms. The patient had a Montreal Cognitive Assessment score of 26 with delayed memory affecting the score the most, although this did not meet pathological criteria. However, further tracking of cognitive capacity is warranted.

We also reviewed the existing literature related to PFBC patients with *MYORG* variants (Table 1). By analyzing this information, we found that patients with dysphagia, verbal impairment, cognitive impairment, and dyskinesia as primary symptoms were mainly middle-aged and elderly (14–18). Conversely, patients with headache as the main clinical symptom are generally younger (10, 19). Therefore, we hypothesize that earlier age of onset is associated with increased risk of headache and migraine, and that other corresponding symptoms may then develop with age.

**Table 1:** Review of the previous literature related to AR-PFBC patients with *MYORG* mutations in comparison to the patients presented in the current report.

**Patient number**	**Sex**	**Age**	**Age at onset**	**Clinical symptoms**	**Cranial CT and cranial MRI**	**Mutations**	**References**
1	Female	12 years	NA	New-onset dizziness, headache, and precocious puberty	Calcification in the lentiform nuclei, bilateral cerebellar white matter, and subcortical white matter in the frontal and parietal regions.	Homozygous mutation: c.856G > A (p.G286S)	(10)
2	Male	52 years	44 years	Recurrent left limb weakness for more than 1 year and exacerbation for 2 days, and headache for 8 years	Cranial CT showed extensive and symmetric calcifications involving the bilateral centrum semiovale, corona radiata, paraventricular, basal ganglia, thalami, vermis cerebelli, cerebellar hemispheres, and midbrain Brain MRI revealed acute ischemic infarction involving the right thalamus	Compound heterozygous mutation: a non-sense mutation (c.1333C > T; p.Q445^*^), and an insertion mutation (c.348_349insCTGGCCTTCCGC; p.R116_S117insLAFR)	(12)
3	Female	45 years	44 years	Dystonia, bradykinesia, dysarthria, dysdiadochokinesia, and irregular postural tremor of her left hand	Symmetric calcification in the bilateral basal ganglion, thalamus, caudate nucleus, red nucleus, and deep and subcortical white matter	Compound heterozygous mutation: c.104 T > A (p.Met35Lys) and c.850 T > C p.C284R	(14)
4	Male	61 years	57 years	Dysphagia and alalia	Multiple symmetric calcifications of bilateral basal ganglia, cerebellum, thalamus, and periventricular area	Compound heterozygous mutation: c.1438T > G mutation and c.1271_1272 TGGTGCGC insertion mutation	(15)
5	Female	54 years	41 years	Extreme fatigue, dysarthria, and movement disorder	Large calcifications affecting the globus pallidus, pons, cerebellum, thalami, centrum semiovale, subcortical white matter, and the cerebral and cerebellar peduncles.	Homozygous duplication: c.854_855dupTG at exon 2 of *MYORG*	(16)
6	Male	43 years	39 years	Progressive cerebellar dysarthria, gait ataxia, dysphagia, forgetfulness, emotional instability, depressive episodes, and intermittent aggressive behavior	Symmetric calcifications in the basal ganglia (pallidum), red nucleus, posterior thalamus, dentate nucleus extending into the cerebellar hemispheres, and periventricular white matter regions and occipital cortex. Hyperintense corticospinal tracts and periventricular microangiopathy were also observed	Homozygous mutation: c.1964A > G p.I655T	(17)
7	Female	45 years	43 years	Mild headaches and cerebellar ataxia including dysarthria	Calcification in the cerebral white matter, basal ganglia, cerebellum, and brainstem	Homozygous mutation: c.794C > T p.T265M	(18)
8	Female	24 years	16 years	Migraine	Symmetrical patchy calcifications in the bilateral cerebellopontine nuclei, thalamus, basal ganglia, and radiocoronal areas	Compound heterozygous mutation: c.580G > T p.Q194^*^ and c.348_c.349insCTGGCC TTCCGC (p.116_ 117insLAFR)	This case report
9	Male	25 years	22 years	Occasional headache	Multiple symmetrical calcifications in bilateral basal ganglia, dorsal thalamus, cerebellar hemispheres, and vermis	Compound heterozygous mutation: c.580G > T p.Q194^*^ and c.348_c.349insCTGGCC TTCCGC (p.116_ 117insLAFR)	This case report

MRI, magnetic resonance imaging; CT, Computed Tomography; NA, not available. ^*^Manifestation of nonsense mutation.

The patient's cranial CT and MRI revealed symmetrical patches of calcified foci in the bilateral cerebellar dentate nuclei, thalamus, basal ganglia, and radiocoronal area, which are typical of PFBC. However, the degree of cerebral calcification in the patient was relatively mild, which may be related to age, disease duration, and mutational pattern. According to previous reports, following onset, the degree of brain calcification in PFBC with *MYORG* gene variants gradually becomes more severe with time (4, 16, 20). Endocrine analysis found normal levels of serum allotropic parathyroid hormone, calcium, and phosphorus. This is also an important point of differentiation between the *MYORG* mutant type of PFBC and AD-PFBC, which involves genes such as *PIT2* (also known as *SLC20A2*) and *XPR1* that are associated with parathyroid regulation of calcium and phosphorus metabolism. Consequently, AD-PFBC is often accompanied by hypoparathyroidism or pseudohypoparathyroidism and intracellular deposition of calcium and phosphorus (5, 7).

**Figure 2:**
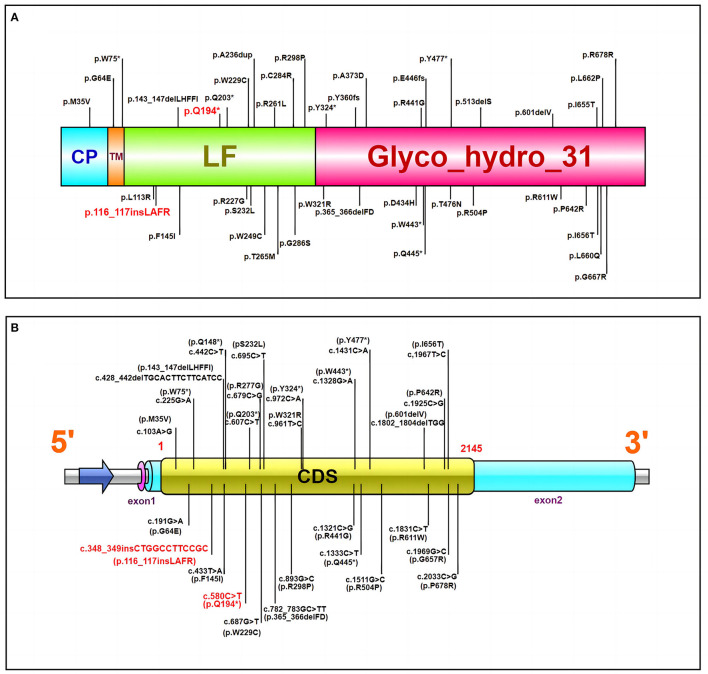
**(A)** The MYORG protein and reported variants. Domain architecture of the MYORG protein and previously reported variants; the variant in our case is marked in red. CP, cytoplasmic domain; TM, transmembrane domain; LF, C-terminal luminal fragment with a glycosidase domain. **(B)** The *MYORG* cDNA and reported variants. The variant in our case is marked in red. CDS Coding sequence (the length is ~2,145 bps). ^*^Manifestation of nonsense mutation.

Whole-exome gene sequencing revealed compound heterozygous mutations on the *MYORG* gene: namely, a non-sense mutation c.580G > T (p.Q194^*^), and an insertional mutation c.348_c.349insCTGGCC TTCCGC (p.116_117ins LAFR). Both mutations are located in the C-terminal tubulin site of *MYORG* (8, 13). We summarized the variants in previous reports (Table 1), including compound heterozygous and homozygous mutations, and identified that the distribution of *MYORG* mutations is mainly segregated in the C-terminal luminal fragment of the *MYORG* protein, which is related to its glycosidase domain (Figure 2). In addition, we found that 9/42 of the mutations we summarized were loss-of-function (LOF) mutations, whereas 27/42 were missense mutations (Figure 2A). *MYORG* is specifically expressed in astrocytes, and may regulate protein glycosylation in the endoplasmic reticulum of brain astrocytes (8, 13, 15). Inactivation of the MYORG glycosidase function may lead to abnormal protein glycosylation and metabolism, which may lay the foundation for the formation of brain calcification (8). Additionally, astrocytes are a key component of the neurovascular unit. Mutations in *MYORG* may cause damage to the neurovascular unit, and accelerate the deposition of calcium and other minerals in small arteries, capillaries, small veins, and perivascular spaces; such neurovascular unit impairment could cause damage to the blood-brain barrier (8, 17). *MYORG* mutations have also been reported in brain hypoperfusion and cerebral infarction (12, 14, 21). However, it has been mentioned in the vascular theory that intracranial vasoconstriction causes migraine aura symptoms, followed by intracranial and extracranial vasodilatation leading to pulsatile headache production. It is also worth noting that, based on multiple recent imaging studies, vascular dilation is not considered to be necessarily present during migraine attacks (22, 23). Furthermore, because the membranes of astrocytes are rich in sodium and potassium pumps, astrocytes maintain a stable K^+^ concentration in the extracellular fluid. Thus, when astrocytes are damaged the electrical activity of neurons may be affected. However, the exact mechanisms by which *MYORG* mutations lead to migraine is unclear, and further studies will help elucidate these mechanisms.

## Conclusion

A patient with a novel mutation in *MYORG* was diagnosed with AR-PFBC at the age of 24 years, with migraine being the only main clinical symptom. Our case highlights the pathogenic profile of the *MYORG* gene and the clinical phenotype of *MYORG* mutations. It also demonstrates the need for exclusion of calcium deposits in the brain for migraine patients with AR inheritance.

## Data availability statement

The original contributions presented in the study are included in the article/supplementary material, further inquiries can be directed to the corresponding author.

## Ethics statement

The studies involving human participants were reviewed and approved by the Ethics Committee of Xiangya Hospital, Central South University. The patients/participants provided their written informed consent to participate in this study. Written informed consent was obtained from the individual(s) for the publication of any potentially identifiable images or data included in this article.

## Author contributions

QX conceived the study. TS drafted the manuscript. TS, YZ, and GW participated in the clinical management of patients and data collection. QX and JD revised the manuscript. QX accepts responsibility for final approval. All authors approved the final version of the article.
